# Bacteria-Mediated Synergistic Cancer Therapy: Small Microbiome Has a Big Hope

**DOI:** 10.1007/s40820-020-00560-9

**Published:** 2021-01-04

**Authors:** Xinyu Lou, Zhichao Chen, Zhonggui He, Mengchi Sun, Jin Sun

**Affiliations:** Wuya College of Innovation, Shenyang Pharmaceutical University, Shenyang, 110016 Liaoning People’s Republic of China

**Keywords:** Bacteria-mediated synergistic cancer therapy, Multiple administration routes, Antitumor responses, Potential side effects, Microbiome approach

## Abstract

The use of bacteria to specifically migrate to cancerous tissue and elicit an antitumor immune response provides a promising platform against cancer with significantly high potency. With dozens of clinical trials underway, some researchers hold the following views: “humans are nearing the first commercial live bacteria therapeutic.” However, the facultative anaerobe *Salmonella typhimurium* VNP20009, which is particularly safe and shows anticancer effects in preclinical studies, had failed in a phase I clinical trial due to low tumor regression and undesired dose-dependent side effects. This is almost certain to disappoint people’s inflated expectations, but it is noted that recent state-of-the-art research has turned attention to bacteria-mediated synergistic cancer therapy (BMSCT). In this review, the foundation of bacteria-mediated bio-therapy is outlined. Then, we summarize the potential benefits and challenges of bacterial bio-therapy in combination with different traditional anticancer therapeutic modalities (chemotherapy, photothermal therapy, reactive oxygen and nitrogen species therapy, immunotherapy, or prodrug-activating therapy) in the past 5 years. Next, we discuss multiple administration routes of BMSCT, highlighting potentiated antitumor responses and avoidance of potential side effects. Finally, we envision the opportunities and challenges for BMSCT development, with the purpose of inspiring medicinal scientists to widely utilize the microbiome approach in patient populations.

## Highlights

- Introducing mechanisms of antitumor activation produced by bacteria-mediated bio-therapy in detail.
- Comprehensively reviewing multiple administration routes of bacterial bio-therapy in combination with different traditional anticancer therapeutic modalities over the recent 5 years.
- Discussing the potential benefits and challenges of this anticancer approach, and conveying the development tendency and the application prospect of this field.

## Graphical abstract

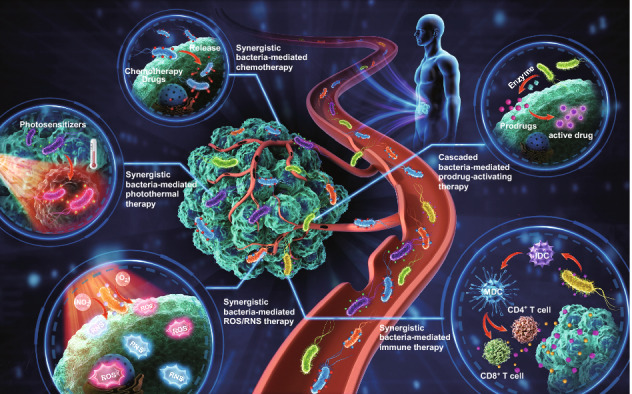

## Introduction

Cancer remains the leading cause of disease deaths all over the world. According to the statistics of the American Cancer Society, cancer resulted in 9.6 million deaths in 2018, accounting for 20% of all deaths [1]. By 2030, it is estimated that there will be 26 million new cases and 17 million cancer deaths worldwide [2]. These statistics underline unprecedented challenges in the treatment of cancer and shed light on the urgency of discovering novel effective antitumor therapies [3]. Conventional anticancer therapies like chemotherapy, radiation therapy, and immunotherapy have been used for cancer treatment. Due to the potential of cancer cells in the generation of resistance to traditional therapies, these treatments have failed to completely eradicate tumor cells [4]. Moreover, the long-term sequelae and side effects significantly impair the therapeutic efficacy of patients treated with traditional therapies.

Here, a recurring concept in our review is bacterial bio-therapy against cancer, which was first recognized a century ago. Bacteria-mediated tumor therapy is long associated with a young surgeon, Dr. William Coley. He might not think that he would be revered as the father of cancer immunotherapy, though he set the stage for a profound bacteria-mediated antitumor treatment in later ages [5]. *Streptococcus pyogenes* capable of preventing cancerous tumor growth was first observed by young Coley, who pointed out that this might be widely put to clinical use [6]. He conducted experimentation and documented proof that several patients diagnosed with end-stage cancers were recovered after they were infected with a mixture of the bacterial species *Streptococcus pyogenes* and *Serrati amarcescens*. The earlier success of Coley’s toxins was an important historical landmark and showed great potential in the cancer therapy. However, being unaware of the concept of cancer immunology at that time, many oncologists questioned the action mode of Coley’s approach. Additionally, for a long time, bacteria-mediated antitumor therapy garnered plenty of doubt due to deadly infections in patients as well as the presence of carcinogenic bacteria (*Helicobacter*, *Salmonella Typhi* and *Fusobacteria*) [7].

This tradition shackle has been broken by scientists’ exploration to determine how to endow specific functions through the advent of synthetic biology approaches and to understand the mechanistic effect of bacteria on human health. The exact mechanism of bacterial colonization in tumor tissues is dependent on the hypoxic, immunosuppressive, and eutrophic tumor microenvironment. With the aid of genetic manipulation, some genetically engineered bacterial strains can secrete cytotoxic products [8]. The tumor-targeting bacteria, as anticancer toxins vectors, can actively migrate to tumor regions, thereby inhibiting cancer growth. Additionally, bacteria can lead to stronger stimulation of immune systems, resulting in the regression of tumors [9]. He et al. reported that p53 and Tum-5-overexpressing *E. coli Nissle* 1917 (EcN) delivers anticancer proteins to tumor. Additionally, engineered bacteria could inhibit the growth of human hepatoma tumors [10]. This previous success indicated that the wide application of bacteria to treat cancers is possible. Moreover, the US Food and Drug Administration (FDA) promulgated guidance on the clinical use of “live biotherapeutic products” with positive expectations [11]. The facultative anaerobe *Salmonella typhimurium* VNP20009 was exploited in the first clinical trial. Specific gene-deleted VNP20009 genetically attenuated the virulence and possessed high safety [12]. The failure of VNP20009 in a phase 1 clinical trial has been sorely disappointing [13]. In view of the trial result, the low tumor responsivity and undesired dose-dependent side effects suggest that a genetic bacteria strategy alone cannot satisfy the practical requirements for high efficiency.

Recent research revealed that a combination of bacterial cancer therapy with other traditional anticancer approaches leads to efficient tumor cell eradication, which could be utilized for overcoming the limitations of monotherapy. BMSCT would have several advantages: (1) possessing a good targeting property; (2) decreasing the side effect via dose reduction; and (3) boosting anticancer immune responses. In this review, we outline the foundation of bacteria-mediated bio-therapy. Then, recent research progress on BMSCT is emphasized for bacterial bio-therapy in combination with chemotherapy, photothermal therapy, reactive oxygen and nitrogen species therapy, immunotherapy, or prodrug-activating therapy. Moreover, multiple administration routes of BMSCT are also discussed in depth.

## Foundation of Bacteria-Mediated Bio-Therapy

### Tumor-Targeting Properties

Live bacteria are capable of specifically “targeting” solid tumors via both passive and active mechanisms. It was assumed that bacteria may initially enter tumor tissue via passive entrapment in the leaky tumor vasculature and then accumulate within the tumor owing to the strong hemorrhage caused by tumor necrosis factor-α (TNF-α) induced by bacterial infection. In the tumor microenvironment, the active scenario likely involves certain chemicals secreted by the dying tumor tissue and hypoxic microenvironment of the tumor tissue [14]. Importantly, the oxygen-enriched environment is detrimental to the survival of anaerobic bacteria, thus further favoring their tumor-targeting specificity. In fact, these mechanisms are not mutually exclusive or strain dependent, and bacteria may use multiple pathways to specifically target tumors [15]. In addition, Forbes et al. have pointed out that specific chemoreceptors and flagella, as well as signal transduction proteins, are necessary for bacteria to target tumors. The researchers analyzed multiple strains with different knock-out cell surface chemoreceptors, or flagella, or signal transduction proteins. These components are found to be essential for the accumulation of *Salmonella typhimurium* in different tumor sites. Among them, aspartate receptors trigger *Salmonella typhimurium* chemotaxis to the tumor site, serine receptors support bacteria penetration, and ribose/galactose receptors make tumor necrosis. In addition, flagella, signal transduction proteins, or active motor functional components are necessary to the directional movement of *Salmonella typhimurium* for effective tumor accumulation [16].

Moreover, the tumor-targeting mechanism of some bacteria highlights the involvement of the immune system. Even though bacteria are willing to home to nutrient-enriched tumors following systemic administration, therapeutic bacteria were observed to similarly disperse in both the tumor and healthy tissues. However, due to the unique immunosuppressive environment generated by tumors, bacteria in normal tissues are eradicated within hours or days, while those in the tumor site could proliferate robustly [17]. Specifically, *Listeria* is known to directly infect not only antigen-presenting cells (APCs), such as macrophages or monocytes and dendritic cells (DCs) [18], but also myeloid-derived suppressor cells (MDSCs) that can then deliver the bacteria to tumor sites. Through this mechanism, *Listeria* residing in MDSCs is protected from immune clearance, while *Listeria* in the healthy tissue milieu is rapidly eliminated [19].

### Mechanisms of Antitumor Immuno-Response Activation

The therapeutic effect of bacteria is mainly due to its immunomodulatory activity [20]. *Salmonella* infection in tumors could lead to antitumor responses by inducing the migration of innate immune cells, including DCs, neutrophils, macrophages as well as neutrophils into colonized tumors [21], and by enhancing the abundant expression of TNF-α, interleukin-1β, and other inflammatory cytokines [22]. Similar to infection by *Salmonella*, the *Clostridial* infection could also lead to the accumulation of macrophages and granulocytes at the infection site, thereby elevating chemokines and cytokines that orchestrate the body’s immune response [23]. Moreover, many bacterial components, such as lipopolysaccharide (LPS), lipoprotein, flagella, and pathogen-associated molecular patterns, play a critical role in the immunostimulatory responses [24]. For example, *Salmonella* LPS could be involved in tumor-specific CD8^+^ T cell responses and the elevation of TNF-α. Flagellin could also significantly suppress tumor cell proliferation and decrease the frequency of CD4^+^, CD25^+^ regulatory T cells [25]. In addition to the innate immune response, bacterial infection also induces adaptive immune responses against tumor cells. In detail, *Salmonella* infection induces the upregulation of connexin 43, which promotes gap junction formation between the tumor cells and adjacent dendritic cells [26]. These functional connections subsequently transfer tumor antigens to DCs and cytotoxic T cells, thus killing tumor cells and preventing metastasis formation. *Listeria* could infect the MDSCs and induce a subpopulation of *Listeria*-carrying MDSCs into an immune-stimulating phenotype characterized by increased production of Interleukin-12, and then enhances CD8^+^ T cell and NK cell responses [27]. Analogously, *Clostridia* could recruit adaptive immune cells including CD8^+^ T lymphocytes to fight against cancer.

### Clinical Research Study of Bacterial Bio-Therapy

There have been a burgeoning number of published studies in recent years on bacteria-based bio-therapy [28], many of which have shown promising prospects in experimental animal models [29]. Nevertheless, these procedures remain experimental, owing to the tumor heterogeneity of cancer patients [30]. It takes tremendous effort to translate microbial therapeutics from the laboratory bench to clinical stages, which involves much ongoing work to regulate and standardize this approach [31]. Spurred by these opportunities, many companies are now utilizing a number of therapeutic modalities [32], such as a genetically modified gut microbiome to target breast cancer and injection of bacteria to treat solid tumors and to re-establish a standardized microbiome [33] (Table 1).

**Table 1:** Clinical trials about bacteria-mediated bio-therapy for cancer treatment

Strain (s)	Cancer type	Mechanism of action	Phase	References
Salmonella Typhimurium (VNP20009)	• Cancer • Neoplasm • Neoplasm Metastasis	Targeting the tumor and infecting cancer cells	Phase I	[34]
Dietary Supplement: Probiotic	• Operable stage I-III breast adenocarcinoma tumors ≥ 1.0 cm	Inducing significant tumor reduction due to the induction of the defense system	Not Applicable	https://www.clinicaltrials.gov/ct2/show/NCT03358511
*Clostridium novyi*-*NT* spores	• Treatment refractory solid tumor malignancies	Infecting tumors and destroying them	Phase I	[35]
MRx0518 a live biotherapeutic product	• Resectable pancreatic cancer	Stimulating the immune function and improving the therapeutic effect of hypofractionated preoperative radiation	Phase I	https://www.clinicaltrials.gov/ct2/show/NCT04193904
ADXS11-001 (ADXS-HPV)	• Head and Neck Cancer • Squamous Cell Carcinoma of the Head and Neck • HPV-Positive Oropharyngeal Squamous Cell Carcinoma	Stimulating the body’s immune system against HPV-positive oropharyngeal squamous cell carcinoma before transoral surgery	Phase II	https://www.clinicaltrials.gov/ct2/show/NCT02002182
APS001F	• Advanced Solid Tumors • Metastatic Solid Tumors	Targeting cancer cells and producing cytotoxic cytosine deaminase (CD)	Phase I/II	https://www.clinicaltrials.gov/ct2/show/NCT01562626
*Clostridium butyricum* CBM 588 Probiotic Strain	• Hematopoietic and Lymphoid Cell Neoplasm	Increasing gut bacteria biodiversity and preventing recurrent symptoms of gastrointestinal toxicity	Phase I	[36]
Typhoid vaccine	• Recurrent Breast Carcinoma • Stage I-IIIA Breast Cancer • Stage I-IIB Breast Cancer	Stimulating the immune system to respond to a tumor	Not Applicable	https://www.clinicaltrials.gov/ct2/show/NCT02415387
JNJ-64041809	• Metastatic castration-resistant prostate cancer	Vaccines for cancer therapy	Phase I	[37]
BacTRL-IL-12	• Solid Tumors	Colonizing solid tumor tissues and delivering genetic material encoding the pro-inflammatory transgene Interleukin-12 (IL-12).	Phase I	https://www.clinicaltrials.gov/ct2/show/NCT04025307

## Bacteria-Mediated Synergistic Cancer Therapy (BMSCT)

Inadequate treatment efficacy often challenges the validity of bacteria monotherapy in cancer treatments. Given the existing conditions, outstanding outcomes are obtained by the combination of bacteria-mediated bio-therapy and other treatments. There are three notable advantages, including: (1) increased antitumor efficacy through synergistic treatment; (2) fewer side effects via tumor-targeting bacteria; and (3) anti-metastases and anti-recurrence by boosting systemic immuno-responses.

### Synergistic Bacteria-Mediated Chemotherapy

To date, chemotherapy is still one of the prominent anticancer approaches against various types and stages of cancer. Despite remarkable recent progress in chemotherapeutic-based delivery systems, chemotherapy is still impeded by off-target side effects and limited intratumoral delivery. Only a minute fraction of i.v.-administered chemotherapeutics distributes to tumor regions through extravasation from blood circulation. Rapid growth of cancer cells, the unusually high fraction of stromal cells, and lack of lymphatic drainage result in accumulated solid stress and elevated interstitial fluid pressure, both of which further diminish the drug supply to tumor sites, preclude tissue penetration, and cause the failure of cancer cell eradication. Given the bacterial tumor-colonizing nature and deep penetration in hypoxic intratumoral regions, bacteria-mediated chemotherapy would overcome the aforementioned problems and improve the therapeutic outcome of chemotherapeutic agents [38].

Bacteria, an effective antitumor drug delivery vector, could conjugate with anticancer drugs via stimuli-responsive linkers. After intravenous administration, drug-loaded bacteria could preferentially translocate to the tumor interstitium and selectively release the drugs in response to the tumor microenvironment, thereby resulting in on-demand drug release. Bacteria-mediated chemotherapy provides a concise strategy to realize temporal and spatial controllability of chemotherapy drugs, showing potential applications in drug delivery.

**Fig. 1:**
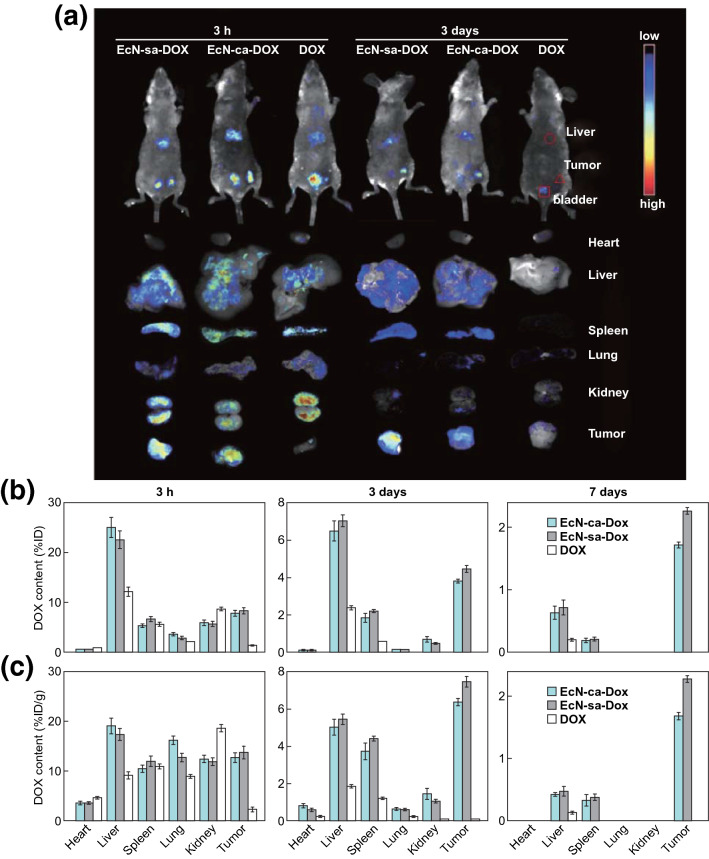
Confirming the tumor-targeting delivery of EcN. **a** *In vivo* fluorescence images of 4T1 tumor-bearing mice and the retrieved organs after intravenous injection of EcN-succinic anhydride-DOX (EcN-sa-DOX), EcN-ca-DOX, and free DOX for 3 h and 3 days. **b** Percentage of injected dose and **c** percent dose rate of DOX in above tissues after injection of EcN-sa-DOX, EcN-ca-DOX and free DOX at a dose of 1 mg DOX Kg^−1^ for 3 h, 3 days and 7 days. Reprinted with permission from Ref. [39]

**Fig. 2:**
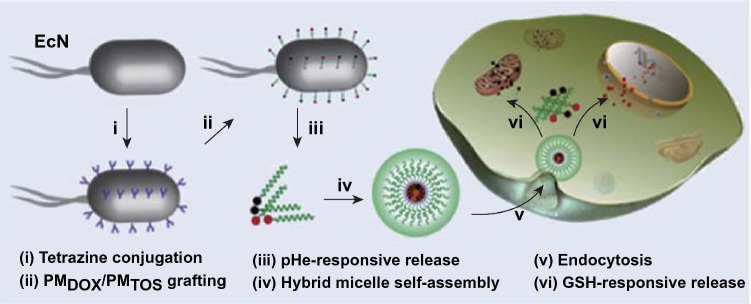
Schematic of bacterial microrobot delivery and release of active drugs for cancer treatment. (1) tetrazine derivatives are conjugated on the surface of EcN; (2) grafting of micellar polymer containing DOX and α-tocopheryl succinate (PM_DOX_/PM_TOS_) by bioorthogonal reactions; (3) the PM_DOX_/PM_TOS_ copolymers are released from the bacterial microbots in response to tumor pHe; (4) PM_DOX_ and PM_TOS_ self-assemble to form hybrid micelles; (5) hybrid micelles are endocytosed into tumor cells; (6) micelles release DOX and α-tocopheryl succinate in response to cytosolic GSH. Reprinted with permission from Ref. [41]

For instance, EcN has been utilized as a bacterial carrier to immobilize doxorubicin (DOX) through acid-labile linkages of cis-aconitic anhydride (EcN-ca-Dox), achieving bacteria-mediated accumulation and pH-responsive release of anticancer drugs in tumor regions [39]. EcN is one of the most studied strains with probiotic potential, which neither secrete toxins nor produce anti-mannose hemagglutinating adhesion. It still maintains good activity after carrying doxorubicin. When the tumor-bearing mice are administered systemically, EcN could proliferate well in solid tumors, but would not accumulate in large amounts in other normal organs. As an amphibian microorganism, EcN could not only remain in the necrotic area of the tumor, but also in the oxygen-rich area, thereby expanding the choice of tumor size and type. More importantly, once an adverse reaction occurs, EcN in the body would be eliminated by antibiotics in time [40]. After intravenous injection, bacteria are rapidly distributed in multiple organs through the circulation, and livers have higher bacteria contents than other tissues owing to the reticuloendothelial system. With time prolonging, bacteria contents in these organs are continuously reduced until cannot be detected and only a few bacteria are found in liver and spleen, suggesting that bacteria are cleared from the body by these organs. Significantly, the fluorescence intensity of bacteria increases in tumor sites with time. This result proves that bacteria possesses inherent tumor-targeting capability and proliferation in vivo. After 3 days injection of EcN-ca-Dox conjugates, the DOX dose amount in tumors was about 5.0% ID g^−1^ tissue, but in the single DOX treatment group, the DOX at the tumor site was almost completely eliminated (Fig. 1). After 30 days injection of EcN-ca-Dox conjugate and DOX into 4T1 tumor-bearing mice, tumors volume in the EcN-ca-Dox group was significantly smaller than those in the Dox group. Moreover, 50% mean survival time of tumor-bearing mice with EcN-ca-Dox group treatment was also about 50% longer than that of Dox group treatment [39]. Compared with free DOX, EcN-ca-Dox treatment improved antitumor effects with respect to the apoptosis induction of tumor cells, tumor growth inhibition, and prolongation of animal survival. Moreover, a novel type of microbot integrating DOX-containing hybrid micelles and self-propelling bacteria has been reported. The pH-responsive release of micelles from bacterial microbots and glutathione (GSH)-sensitive intracellular release of DOX resulted in significant antitumor efficacy with low toxicity (Fig. 2) [41].

In addition, bacteria-derived minicells are also suitable candidates for the encapsulation of chemotherapeutics [42]. It was reported that minicells could pack a range of chemotherapeutic drugs despite their disparate charge, solubility, and hydrophobicity [43]. Scientists have become aware that drug resistance in cancer chemotherapy is a bitter headache [44]. Given the positive effects of minicells loaded with small interfering RNA (siRNA) in terms of their ability to knock down multidrug resistance proteins and increase tumor sensitivity to chemotherapy, MacDiarmid et al. demonstrated that subsequent administration of targeted minicells containing siRNA and cytotoxic drugs showed significant antitumor activity, which led to a several thousand-fold reduction in the dose of chemotherapeutic drug and shortened the period of chemotherapy [45].

However, there are also some limitations and challenges in the synergistic bacteria-mediated chemotherapy. The chemotherapy drugs could shuttle directly to the cancer tissue under the transportation of the carrier bacteria. The drug release from the carrier bacteria is the key to eliminate the tumor. Thus, the chemotherapy drugs must be stably loaded on the surface of the carrier bacteria in systemic circulation, but can be effectively released at tumor sites. The simply electrostatic interaction between drugs and carrier bacteria is insufficient for stability. However, drugs are directly connected to the surface of the bacteria through covalent interaction such as amide bonds, causing slow drug release at the tumor site. Meanwhile, applying synergistic bacteria-mediated chemotherapy to treat the drug-resistant tumors is another challenge as an ideal balance between therapeutic benefit and pathogenicity. For the drug-resistant tumors, chemotherapy drugs delivered by bacteria cannot achieve the desired therapeutic effect, and the carrier bacteria are required to play a therapeutic role. However, therapeutic bacteria might face safety problems due to toxic side effects on normal tissues [46].

In the future, researchers need to spend more time in designing a reasonable drug delivery system of synergistic bacteria-mediated chemotherapy to ensure that the agents precisely reach the tumor site and could be released on demand. For example, the linker between drugs and carrier bacteria should continue to be explored so that drugs could be released from the carrier bacterial in response to acidic, redox and hypoxic tumor microenvironment. We also believe that an optimized balance could be achieved to treat drug-resistant tumors through genetic engineering and more reasonably attenuate bacteria-induced side effects.

### Synergistic Bacteria-Mediated Photothermal Therapy

Photothermal therapy (PTT) uses photosensitizers (PSs) with specific light absorption in the near infrared to convert light energy into heat energy to kill target cells. In bacteria-mediated PTT therapy, PSs were attached to the surface of bacteria to develop a bacteria-driven PSs delivery system for tumor precision therapy. Bacteria could retain its viability after efficiently linking with PSs and pervade throughout the solid tumor with its self-driven properties. Under near-infrared laser irradiation, PSs-coated bacteria exhibited good cancer cells killing ability for eradicating the solid tumor. This combined approach has a great value for solid tumors therapy with high efficiency and low toxicity [47].

**Fig. 3:**
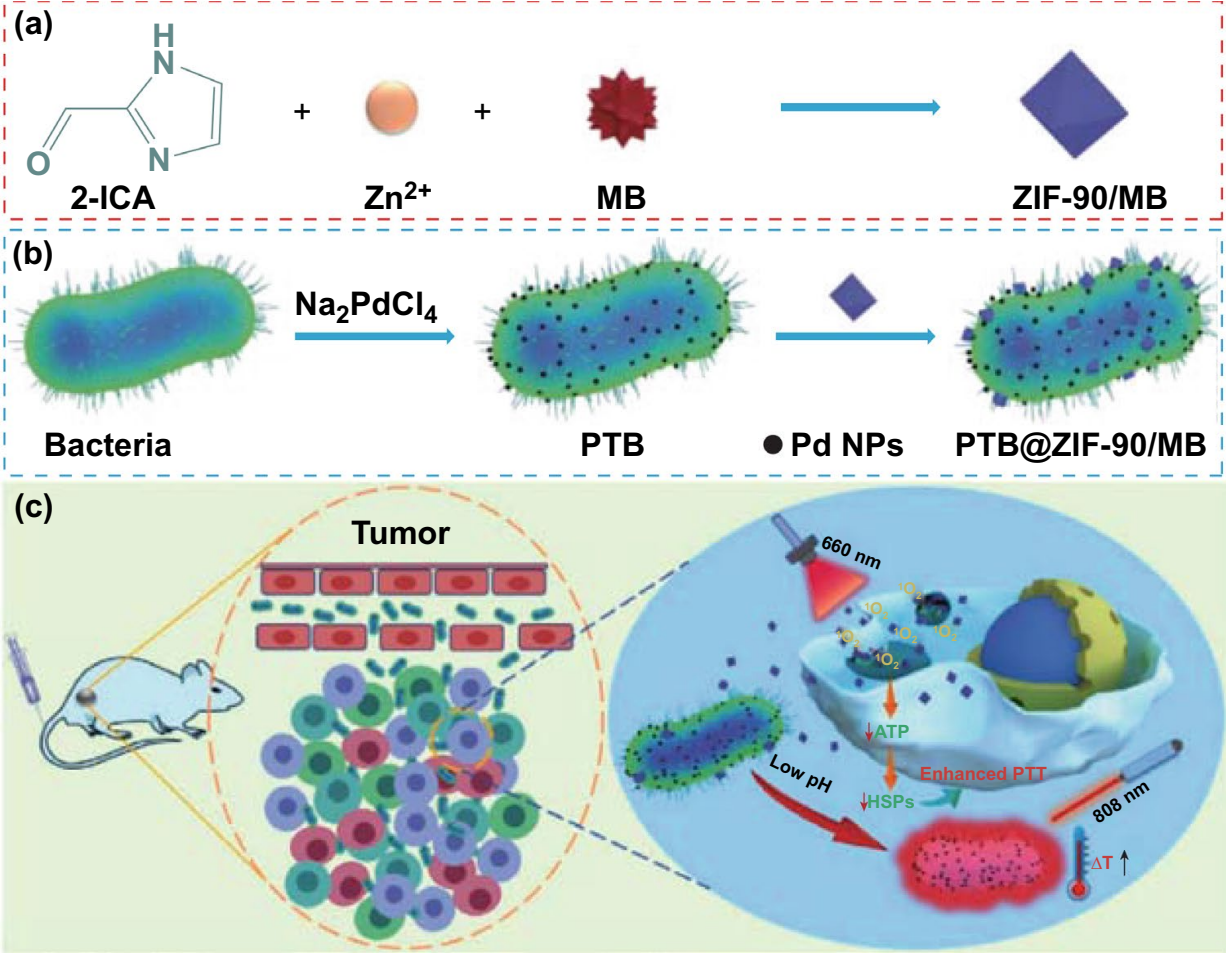
Schematic of the PTB@ZIF-90/MB formation process and antitumor mechanism. **a** Synthetic procedure of photosensitizer methylene blue encapsulated zeolitic imidazole frameworks-90 (ZIF-90 MB). **b** Electrochemically active bacterium *Shewanella oneidensis* MR-1 reduces sodium tetra-chloropalladate (Na_2_PdCl_4_) into palladium nanoparticles (Pd NPs) on bacterial cell surface to convert to photothermal bacterium (PTB),then ZIF-90/MB was conjugated on the surface of PTB to obtain PTB@ZIF-90/MB. **c** After PTB @ ZIF-90/MB reaches the acidic tumor site, ATP induces the release of MB, and further produces ROS under light to enhance the photothermal treatment effect of PTB. Reprinted with permission from Ref. [51]

Recent studies on bacteria-mediated PTT have shown their high value in the cancer therapy. Chen et al. attached the INPs (PSs-containing indocyanine green (ICG)) to the surface of YB1 (a safe *Salmonella Typhimurium* strain) via amide bonds to develop a biotic/abiotic complex (YB1-INPs) for anticancer drugs. Based on the specific tumor hypoxia-colonizing nature of YB1, the YB1-INPs achieved effective tumor targeting, eliminated the tumors in mice after NIR laser irradiation, and also showed satisfying fluorescence (FL) imaging ability. After intravenous injection of INPs or YB1-INPs into C57BL/6 mice with transplanted MB49 tumors, the researchers used an in vivo imaging system to monitor the ICG FL of the animals. Within 72 h, there were no obvious FL signals in the tumor site of the mice in the ICG group due to the lack of the targeting ability. However, obvious FL signals were detected in the YB1-INPs group because the YB1 carrier could target hypoxic regions of the tumors. Next, 12 h after the INPs and YB1-INPs treatment, the researchers irradiated each tumor-bearing mice group. They found that the temperature of the tumor region in the INPs group was only 43 °C not enough to eliminate the tumor, while the temperature of the tumor in the YB1-INPs group reached up to 63 °C. Therefore, the tumor inhibition rate was 100% at 28 days under the YB1-INPs treatment, and even more exciting the survival rate was 100% at the end of the experiment. But in the INPs group, we did not observe significant tumor suppression. All in all, the strategy of synergistic bacteria-mediated PTT has greatly improved ICG targeting and tumor treatment effects [48]. In addition, VNP20009 can also target to the tumors because of its preference for hypoxia in the core of the tumor and necrotic tumor tissues. Some specific genes had been deleted in VNP20009, which could notably reduce its toxicity, strengthen safety, and the risk of septic shock. Moreover, there are a large number of preclinical and clinical results for researchers to learn [49]. By leveraging the self-polymerization phenomenon of dopamine (DA), the surface of the facultative anaerobe *Salmonella strain* VNP20009 was enveloped with polydopamine. After intravenous injection, a large quantity of polydopamine on the surface of VNP20009 was accumulated in the tumor tissues, achieving sufficiently high temperatures to kill tumor cells with less damage to other organs [49]. Naturally, certain bacteria can grab environmental metal ions and translate these ions into the elemental metal by biomineralization methods without affecting bacterial variation [50]. Inspired by this phenomenon, the Zhang group reported that palladium nanoparticles (Pd NPs) were biomineralized on the surface of the facultative anaerobic bacterium *Shewanella oneidensis* MR-1 (*S. oneidensis* MR-1). As shown in Fig. 3, this bacteria-based photothermal therapeutical platform was found to possess great tumor-targeting features and increased photothermal capacity under near-infrared (NIR) laser irradiation [51].

Different delivery approaches have different effects on therapeutic efficiency. In a comparison study conducted by Luo and co-workers, two strategies for connecting bacteria with PSs through electrostatic interactions and antibody-directed action were prepared. The first method utilizing electrostatic interaction is called the cargo-carrying method, and ligand-free core–shell structural upconversion nanoparticles (LF-UCNPs) were electrostatically deposited on the vegetative B. *breve* bacterial surface with negative charge for good delivery into the tumor region. The other is the antibody-directed method, in which core–shell structural upconversion nanoparticles (CS-UCNPs) were wrapped with a silica shell and then linked with Clostridium polyclonal antibodies via PEG polymers for migrating and binding to the tumoral vegetative C. *difficile*. The in vitro and in vivo experimental results confirmed that the antibody-directed method could accumulate more nanoparticles to hypoxic tumor tissues and produce enhanced higher PTT-mediated antitumor effects compared with the cargo-carrying method [52].

PSs wrapped with bacteria-derived outer membrane vesicles (OMVs) as a delivery system have also aroused great interest. OMVs possess many advantages such as safety, stability, and easy modification. Melanin can convert 99.9% of the absorbed light energy into heat, possessing good PTT-mediated antitumor efficacy [53]. Gujrati et al. successfully engineered OMVs encapsulating melanin (OMV_Mel_) to target tumor tissue for diagnosing and ablating tumors [54]. Moreover, to improve OMVs’ functionality and expandability, Chen et al. proposed a eukaryotic–prokaryotic vesicle nanoplatform, that is, melanoma cytomembrane vesicles (CMVs) and attenuated *Salmonella* OMVs were fused and wrapped with PS indocyanine. This nanoplatform integrates melanoma antigens with PTT to enhance the therapeutic effects of antitumor vaccination [55]. After much efforts, it was determined that bacteria-derived OMVs would be sensed and ingested by neutrophils in vivo [56]. The OMVs hitchhike neutrophils in situ, achieving the effective migration and penetration of inflamed tumors [57]. Based on the specific targeting mechanism, Li et al. designed PTT transducer-obtained nanoparticles coated with OMVs that could be internalized by neutrophils to improve photothermal treatment efficiency. Compared to traditional synthetic nanoparticles (NPs) delivered solely by controversial passive targeting, i.e., the enhanced permeability and retention (EPR) effect, the innovative OMV-induced mimicking delivery strategy holds high promise for improved nanomedicine delivery in tumor therapy [58].

Prior to therapeutic effect, the safety of this therapy is still a challenge we need to face in the synergistic bacteria-mediated PTT. Several studies have found that after applying PTT to tumor-bearing mice, the weight of the mice was reduced compared with the control group. This phenomenon has demonstrated that this therapy has certain side effects, and the reasons were various. For example, the PSs selected have certain toxicity, and bacteria might cause some inflammations and affect normal tissues and organs. For OMVs delivery system in the synergistic bacteria-mediated PTT, the tumor-targeting efficiency could be insufficient. Meanwhile, since OMVs lack flagella and other motor elements, it is difficult to penetrate into the tumor core, so OMVs-mediated PTT would not completely eliminate the deep cancer cells. Moreover, as for the large-scale production, many difficulties such as sterilization, drug loading, and stability need to be overcome [53].

In the future, we must fully evaluate the safety of bacteria or bacteria-derived OMVs, ensuring the biocompatibility of the delivery system and avoid acute inflammation or autoimmune diseases. For PTT systems using bacteria-derived OMVs, the modification of tumor-targeting ligands on the surface would endow OMVs excellent tumor targeting, making them effective against cancer cells. With the invention of new materials and the formation of new quality evaluation standards, synergistic bacteria-mediated PTT therapy would have broad prospects [58].

### Synergistic Bacteria-Mediated Reactive Oxygen and Nitrogen Species (ROS/RNS) Therapy

Disturbed redox homeostasis, as a hallmark of cancer phenotypes, influences the development of tumors [59]. The cancer cells usually show high levels of reactive oxygen and nitrogen species (ROS/RNS), such as superoxide (O_2_^·^), hydrogen peroxide (H_2_O_2_), hydroxyl- (^·^OH), and nitric oxide (NO) [60]. ROS/RNS play a distinct dual role, entailing protumorigenic and tumor inhibitory effects at different intracellular concentrations, respectively [61]. The cancer cells keep steady-state ROS/RNS levels within a narrow range, allowing for rapid growth and invasion, while cancer cells can be directly killed under higher ROS/RNS concentrations [61]. Herein, bacteria carrying ROS/RNS generation enzymes are combined with nanocatalysts for achieving bacterial metabolite therapy. In this constructed bioreactor, bacteria could effectively accumulate in tumors and sustainably synthesize ROS/RNS with the aid of nanocatalysts, thus resulting in severe tumor apoptosis.

**Fig. 4:**
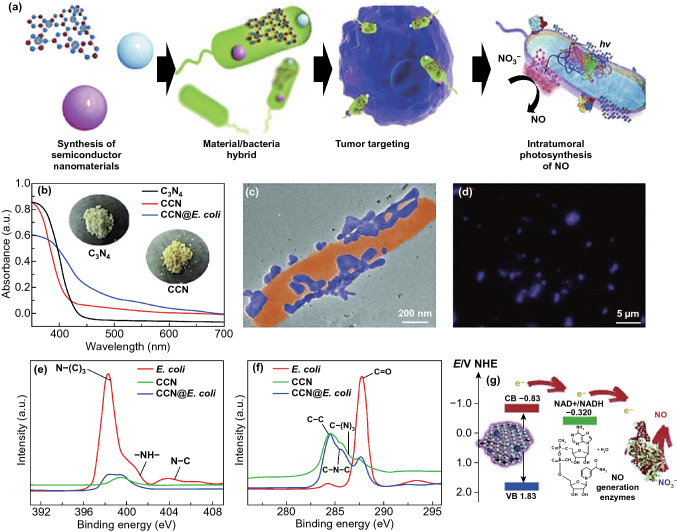
Characterization of a bacteria-mediated reactive nitrogen species therapy system. **a** Schematic of CCN@*E. coli* for photo-controlled bacterial metabolite anticancer treatment. First, carbon-dot-doped carbon nitride (CCN) with suppressed free radical generation capability was synthesize. Furthermore, CCN and *E. coli* MG1655 (*E. coli*) were assembled through electrostatic interactions to obtain CCN@*E. coli*. Then, CCN@*E. coli* can be targeted to the tumor site and metabolize NO_3_^−^ to antitumor NO under photo-irradiation. **b** UV–Vis absorption spectra of the CCN. **c** TEM image of CCN@*E. coli*. **d** Spinning disk confocal microscope image of CCN@*E. coli* (Red: *E. coli*, Blue: CCN). **e** XPS de-convoluted spectra for the N1s orbitals of *E. coli*, CCN, and CCN@*E. coli*. **f** XPS de-convoluted spectra for the C1s orbitals of *E. coli*, CCN and CCN@*E. coli*. **g** Schematic illustration for the photoelectron transport among CCN, electron acceptor and NO generation enzymes. Reprinted with permission from Ref. [62]

Zheng and co-workers reported a strategy in which *E. coli* MG1655 were charged with a nano-photocatalyst to promote their metabolic activities for achieving photo-controlled bacterial metabolite anticancer treatment [62]. *E. coli* MG1655 has excellent tumor colonization ability and can targetedly deliver antitumor nanomaterials. In addition, *E. coli* MG1655 possess the capacity of driving intracellular reaction at the expense of exogenous electrons, so that it can drive intracellular reactions, enhance metabolic activity, and stimulate potential anticancer effect in the presence of external electrons [62]. First, they synthesized carbon-dot-doped carbon nitride (CCN) with an inhibitory effect on the generation of free radicals to achieve in situ photoelectric conversion. Subsequently, CCN is assembled with *Escherichia coli* (*E. coli*) carrying nitric oxide (NO) generation enzymes via electrostatic interactions to obtain CCN@*E. coli*. Under light irradiation, abundant exogenous electrons produced by carbon nitride could be transferred to *E. coli* MG1655 to strengthen the enzymatic reduction of endogenous NO_3_^−^ to antineoplastic NO. These cytotoxic NO molecules increased by 37-fold, exhibiting strong anti-proliferative activity in vivo. Their experiments proved that the CCN@*E. coli* group had a good inhibitory effect in both 4T1 and CT26 tumor-bearing mice, with the inhibition rate of more than 70% due to the high level of NO in the tumor site. But the tumor suppression effect by the CCN group was very poor (Fig. 4) [62].

**Fig. 5:**
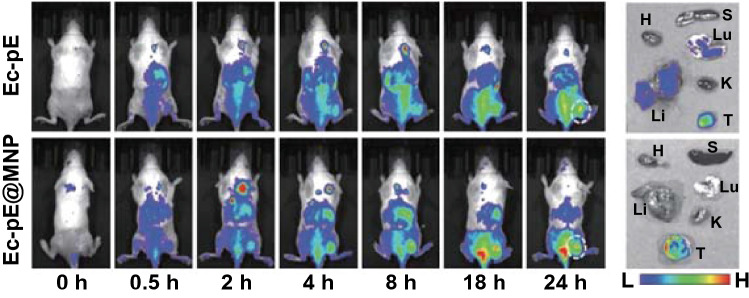
Optical images of 4T1 tumor-bearing mice and major organs after intravenous injection of *E. coli* MG1655 with NDH-2 enzyme overexpressed (Ec-pE) and Fe_3_O_4_ nanoparticles decorated Ec-pE. Reprinted with permission from Ref. [63]

Recently, therapeutic Fenton-like reactions for generating toxic ROS have received increasing attention. One example is the use of a Fenton-like bioreactor composed of magnetic Fe_3_O_4_ nanoparticle-decorated *E. coli* MG1655 overexpressing respiratory chain enzyme II (NDH-2) [63]. In this constructed bioreactor, the Fenton-like reaction occurs with continuously generated H_2_O_2_ produced by engineered bacteria, and abundant tumor cell apoptosis is triggered by the produced cytotoxic hydroxyl radicals (^·^OH). Certain bacteria-based self-supplied therapeutic Fenton-like bioreactors offer a promising approach to eliminate tumor cells and have shown great potential in biomedical fields (Fig. 5) [63].

**Fig. 6:**
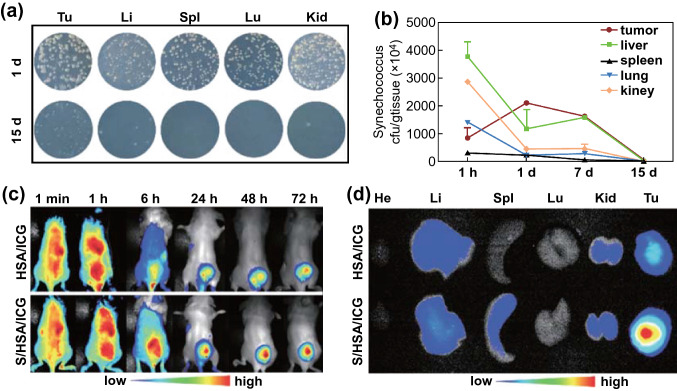
**a** Bacterial colony-forming units of tissue homogenates in BG11 solid plates after intravenous injection of HSA/ICG-loaded Syne for 1 and 15 days. **b** Bacteria growth profiles of different tissues within 15 days. **c** 4T1 tumor-bearing mice and **d** major organs after intravenous injection of indocyanine green-encapsulated human serum albumin nanoparticles and HSA/ICG NPs-loaded photosynthetic bacteria. Reprinted with permission from Ref. [65]

**Fig. 7:**
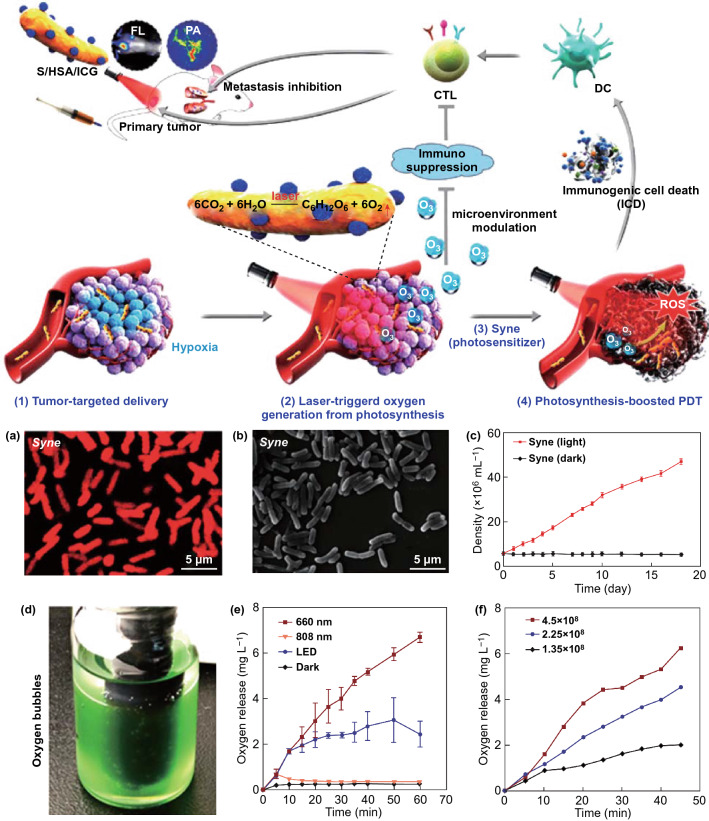
Schematic of nanophotosensitizer (HSA/ICG) conjucated Syne as a photocatalyzed oxygen generation system (S/HSA/ICG) for metastatic tumor immunogenic PDT. S/HSA/ICG can target to the tumor site due to the tumor-targeting abilities of HAS and Syne. At the tumor site, Syne produced a large amount of oxygen and promote the production of ROS under laser radiation. Meanwhile, the oxygen can ameliorate tumor hypoxia and reverse the tumor immunosuppressive microenvironment, and further elicit the immunogenic cell death-mediated antitumor immune response to enhance the PDT efficacy. **a** FL micrograph of Syne. **b** Scanning electron micrograph of Syne. **c** Growth curve of Syne. **d** Bubbles generated in the Syne suspension under light. **e** Oxygen release curves of Syne of various conditions. **f** Oxygen production curves of different concentrations of Syne under 660 nm laser irradiation. Reprinted with permission from Ref. [65]

As a common and feasible ROS approach, photodynamic therapy (PDT) has been extensively used in the treatment of cancers of various locations and types, because of its availability, efficiency, noninvasive properties, and good tolerance. It relies on PSs to convert oxygen into cytotoxic ROS under light irradiation of a specific wavelength to ablate malignant tumors [64]. However, the delivery of PSs is still a challenging research topic due to failure to reach the tumor tissue. To address the problem of the tumor-targeting direction of PSs, bacterial cancer therapy has been investigated in combination with PDT in an experimental tumor model with progressive results. Additionally, hypoxia in the tumor microenvironment is another hurdle dampening the anticancer effect of PDT. Advanced bacterial therapy has been developed to ameliorate tumor hypoxia via increasing the oxygen concentration in the tumor. Liu et al. reported that *Synechococcus* 7942 with tumor-targeting and oxygen-carrying dual function was used to deliver the ICG to 4T1-bearing breast tumors, which significantly suppressed tumor growth and inhibited pulmonary metastasis. In the study, human serum albumin (HSA) and ICG were combined through intermolecular disulfide to construct the nano-PSs (HSA/ICG). Next, the HSA/ICG was modified on the surface of *Synechococcus* 7942 to form S/HSA/ICG via amide bonds. Then researchers compared the tumor-targeting and antitumor effects of S/HSA/ICG and HSA/ICG. Tumor-bearing mice were injected intravenously with HSA/ICG or S/HSA/ICG. The ICG FL signal monitored by the in vivo imaging system, 72 h after administration, showed that S/HSA/ICG showed more ICG FL signal in the tumor compared with HSA/ICG (Fig. 6). Moreover, after laser irradiation, the tumors in the S/HSA/ICG group were completely ablated, but tumor volumes in the HSA/ICG group exceeded 1000 mm^3^ within 24 days. These results had shown that the bacteria-mediated synergistic application greatly improved the therapeutic effect (Fig. 7) [65]. Except for the direct therapeutic effect, co-administration of bacteria and PSs could also be utilized to realize effective protein and gene release. It has been discovered that light-induced generation of ROS from PSs on the surface of *E. coli* could damage the bacterial membrane and thus achieve controllable release of toxic proteins. This work could achieve effective light-mediated protein delivery [66].

In the bacteria-mediated ROS/RNS therapy, therapeutic efficiency has aroused our attention. Some ROS/RNS therapy systems require the participation of bacteria to produce ROS/RNS concurrently, so it is necessary to maintain the genetic stability. Under irradiation, bacteria may undergo genetic mutations so that bacteria cannot produce ROS/RNS and lose their therapeutic effect. At the same time, due to the poor penetration of radiation, the ROS/RNS generation efficiency at the deep tumor site would be decreased, so the treatment of deep tumors needs more irradiation. However, the excessive irradiation could damage the skin tissue and cause side effects [62].

Bacteria-mediated ROS/RNS therapy could effectively eliminate tumors by producing ROS or RNS simultaneously. It could also effectively improve the tumor immunosuppressive microenvironment and strongly stimulate the system’s antitumor immune response, showing excellent effects on tumor recurrence and metastasis suppression. This provides a promising strategy against cancer. In the future, in addition to find the safe and tumor-targeting bacteria, we should further design effective PSs which could eliminate deep tumors with fewer irradiation.

### Synergistic Bacteria-Mediated Immune Therapy

**Fig. 8:**
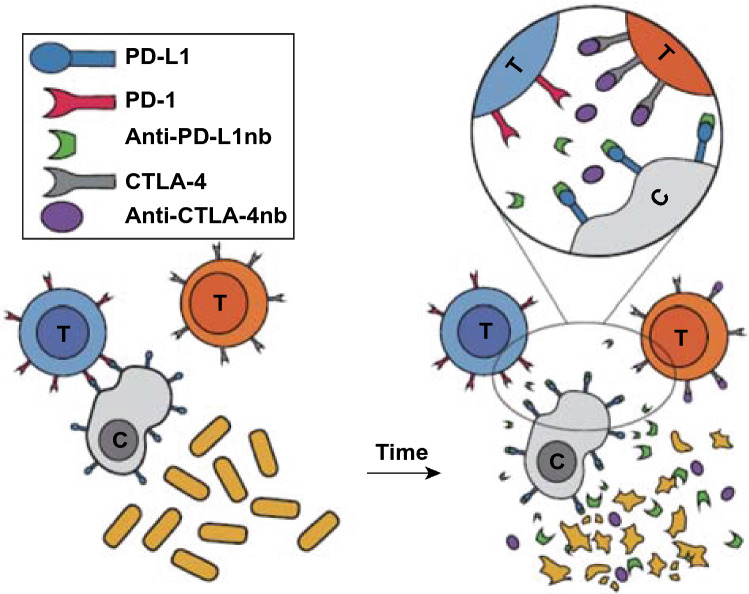
Schematic diagram of engineered bacteria delivering immune checkpoint inhibitors. Engineered bacteria carrying immune checkpoint inhibitors gather in the core of the tumor, grow to a critical density and lyse, thereby continuously releasing therapeutic agents at the tumor site to treat cancer. Reprinted with permission from Ref. [69]

The immune system is a complicated defense network of life to maintain homeostasis in the body. Immune therapy was recently recognized as an emerging and promising strategy for cancer treatment, which concentrates on leveraging the immune system’s ability to attack tumor cells through immune stimulation of cancer patients. Although many strategies have induced measurable immune responses, only a small number of treated patients have shown clinical benefit. Because of the unfavorable therapeutic responsibility of current cancer immunotherapy strategies, more adjuvant vectors, formulations, and new immunogenic antigens are undoubtedly needed. Mounting studies suggested the efficacy of bacterial immunity against solid tumors. For example, the engineered bacteria-mediated antigen delivery is a promising strategy for cancer treatment. After intravenous injection, antigen-secreting engineered bacteria could colonize tumor tissues and induce the infiltration of immune cells. Subsequently, the antigen secreted by colonizing bacteria would lead to the activation of intratumoral T cells to attack tumor cells. This strategy could effectively shape the host antitumor immune response and significantly suppress the growth of tumor. The *E. coli* TOP10 is another such example that can induce significant tumor reduction in a colon carcinoma mouse model due to the induction of tumor-specific CD4+ and CD8+ T cells. In the induction phase, CD8+ T cells were the sole effectors in immunological responses against tumors, while CD8+ as well as CD4+ T cells were involved in the memory phase [67]. Additionally, it was demonstrated that CD47-encoding *E. coli* organisms could stimulate systemic tumor antigen-specific immune responses, induce durable tumor regression, and lead to long-term survival in a syngeneic tumor model [31]. Furthermore, attenuated live strains of *Salmonella typhimurium* have been used to secrete a broad range of tumor antigens. The expression of oncogenes such as HPV16-E7, CEA-scFv, and Vibrio vulnificus flagellin B helps to stimulate the immune response [68]. A vaccine strain of *Salmonella typhimurium* expressing NY-ESO-1 tumor antigen showed the effect of tumor regression. Animals intravenously administered with *S.typhimurium* NY-ESO-1 displayed CD8+ T cell responses [9]. At the same time, engineered probiotics can also be used to deliver checkpoint blocking nanobodies, such as the cytotoxic T lymphocyte–associated protein-4 (CTLA-4) and programmed cell death–ligand 1 (PD-L1) to improve antitumor efficacy (Fig. 8) [69].

Another convenient strategy is the use of OMVs that can effectively induce long-term antitumor immune responses for cancer immunotherapy and load drug to kill cancer cells directly. The OMVs are naturally produced from Gram-negative bacteria during their normal growth and have nano-sized (20–250 nm) lipid bilayer structures composed of various immunogenic components. A recent study indicated that systematically administered OMVs could promote the production of the antitumor cytokines interferon-γ and CXCL10 to activate a strong antitumor response [70]. Furthermore, compared with live or attenuated bacteria, OMVs are considered to be safer owing to their non-replicative nature. Therefore, they are considered as ideal candidates and promising carriers for tumor-targeted drug delivery carriers. Bioengineered OMV-coated polymeric nanomedicines have been reported to effectively inhibit tumor growth and provide protective immunity against tumor occurrence [71]. In detail, OMVs of attenuated *Salmonella typhimurium* were functionalized with polyethylene glycol (PEG) and Arg-Gly-Asp (RGD) peptide to improve their colloidal stability and tumor-targeting capacity. Tegafur was selected as a candidate drug for chemotherapy due to its antineoplastic effect and ability to synergistically facilitate the immunostimulatory capacity of OMVs. Collectively, these functionalized OMV-coated polymeric nanomedicines could dramatically boost the efficacy of cancer immunotherapy [71].

Bacterial membrane, characterized by abundant pathogen-associated molecular patterns, demonstrated strong antitumor activity by stimulating innate immunity and dendritic cell (DC) activation [72]. It has been shown that bacterial membrane-coated nanoparticles composed of immune activating PC7A/CpG polyplex core can capture cancer neoantigens following radiation therapy, facilitate their uptake in DC cells, stimulate an antitumor T cell response, and thus result in tumor regression and specific antitumor immune memory. In detail, Chen et al. designed a OMVs-coated Tegafur (FT)-loaded polymeric micelles (ORFT) with the DSPE-PEG-RGD modification on the surface. In their study, effector memory T cells were significantly higher under the ORFT group treatment, and both IFN-γ and IL-12 significantly enhanced compared with the FT group. At the end of the experiment, the tumor volumes of the mice in the ORFT group were only half that of the FT group. Moreover, 20% of the mice in the ORFT group survived for up to 39 days while the mice in the FT group survived for no more than 31 days. These results provide the evidence that functionalized OMVs, in combination with immune treatment, could effectively activate the immune system to treat the tumor [72].

The clinical applications of bacteria-mediated immune therapy encounter some challenges associated with efficacy and safety. The bacteria-mediated immune therapy depends on identifying specific defects or dysfunctions in the antitumor immune response. However, tumor-induced immune defects possess high heterogeneity. This heterogeneity not only exists among different patients, but also occurs in a single tumor lesion. Thus, only a small number of patients respond to immunotherapy and devastatingly subject to relapse. Regarding the safety, immune cells are equally effective to clear tumor and normal cells when the target antigen is shared by tumor and normal tissues leading to serious adverse effects in major organs.

Solving the challenges related to the efficacy and safety of bacteria-mediated immunotherapy is still a central task for the future research. Discovering novel immune checkpoint inhibitors, genetically engineered bacteria with excellent immunotherapy effects, and OMVs delivery systems with tumor targeting, might be important means to solve above challenges [73].

### Cascaded Bacteria-Mediated Prodrug-Activating Therapy (CBPT)

With the exploration and development of chemotherapy against cancer, the dose-dependent toxicity of chemotherapeutics has become a thorny issue. Cascaded bacteria-mediated prodrug-activating therapy, which can reduce the dose of chemotherapeutics to decrease the toxicity of chemotherapy, has attracted researchers’ attention [74]. CBPT, referred to as in situ chemotherapy, combines bacteria and prodrugs to cure cancer. First, therapeutic bacteria, mostly natural probiotics, colonize and multiply at the tumor site and then secrete the sufficient levels of prodrug-converting enzymes. Next, antitumor prodrugs are administered and distributed to the whole body, and then are converted into antineoplastic drugs under the action of the special bacteria-produced prodrug-converting enzymes [75]. For instance, *Bifidobacterium breve* and *Lactococcus lactis* can activate CB1954 (Tretazicar), *Lactobacillus* species can activate the prodrug of 5-fluorocytosine, and *E. coli Nissle 1917* can activate a variety of prodrugs including AQ4N, CB1954, tegafur, fludarabine phosphata and 5-fluorocytosine. In tumor-bearing mice, the combination of probiotics and prodrugs can significantly inhibit tumor growth and improve the survival rate [76].

In CBPT, it is not only the combination of prodrugs and probiotics but the combination of prodrugs and engineered bacteria with the ability to express specific prodrug-converting enzymes, which can further exert an antitumor effect. Engineered bacteria with genes encoding specific prodrug-converting enzymes more effectively transform the prodrugs into drugs with cytotoxicity. Among them, engineered bacteria combined with glucoside prodrugs have an important application. For example, engineered *E. coli* expressing glucuronidase on the bacterial surface can promote the hydrolysis of p-hydroxy aniline mustard β-D-glucuronide to produce the active drugs p-hydroxy aniline mustard. In vivo experiments have shown that the combined application of engineered *E. coli*-mediated prodrug-activating therapy can enhance the antitumor effect and reduce toxicity and side effects to a large extent since the content of β-glucuronidase is low in the normal tissues [77].

**Fig. 9:**
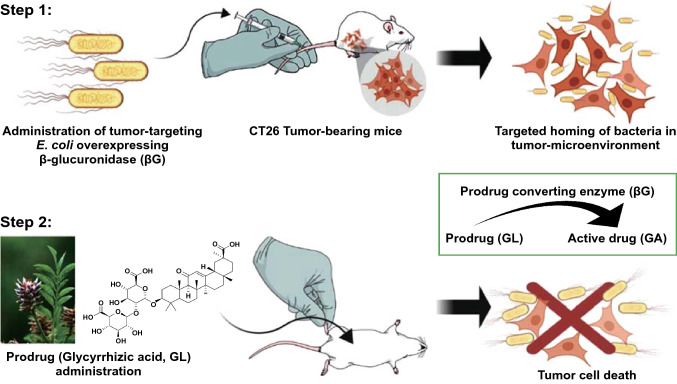
Schematic of engineered *E. coli* overexpressing β-glucuronidase (βG) combined with prodrug (glycyrrhizic acid, GL) for tumor treatment. Step 1, engineered *E. coli* overexpressing β-glucuronidase (βG) is injected intravenously into a tumor-bearing mice model and then colonizes in the tumor site. Step 2, GL is activated by βG into a cytotoxic antitumor drug (glycyrrhetinic acid, GA) at the tumor site to eliminate the tumor. Reprinted with permission from Ref. [80]

The choice of prodrug is also important in CBPT. The prodrugs must not only have little adverse effect on the normal tissues but only a slight influence on the probiotics or engineered bacteria expressing prodrug-converting enzymes [78]. Natural and nontoxic compounds should be used more frequently rather than synthetic prodrugs, thereby further simplifying the operation and improving the safety of CBPT. Recent studies found that natural glycyrrhizic acid could be catalyzed by β-glucuronidase into glycyrrhetinic acid (GA) with strong cytotoxicity [79]. Combination treatment with *E. coli* expressing β-glucuronidase and glycyrrhizic acid showed a good tumor suppression rate with a low toxicity in a colon cancer mouse model (Fig. 9) [80].

It is extremely important for bacteria to target the tumor site and avoid the enzyme-mediated converted production in normal tissues. If bacteria expressing prodrug-converting enzymes are distributed in normal tissues and multiply in large numbers, large amounts of prodrug-converting enzymes could transform prodrugs into cytotoxic drugs in normal tissues and cause serious side effects. Moreover, only engineered bacteria with stable genetic expression could continue to produce prodrug-converting enzymes at the tumor site. However, studies have found that engineered bacteria stimulated by the external environment might induce mutations and affect the production of prodrug-converting enzymes [81].

In the future, it is necessary to select engineered bacteria with good tumor targeting for cascaded bacteria-mediated prodrug-activating therapy. At the same time, engineered bacteria would be rationally designed, which could intelligently control gene expression for antitumor therapy. As such, we could design the engineered bacteria with gene expression under the specific laser irradiation. We should also choose safe prodrugs to avoid affecting normal tissues and minimize the side effects in the cascaded bacteria-mediated prodrug-activating therapy [81].

## Multiple Administration Routes of Bacteria-Mediated Synergistic Cancer Therapy (BMSCT)

### Oral Administration Route of BMSCT

As mentioned above, during the development process of BMSCT, intravenous injection (i.v.) or intratumoral injection is most commonly used. At the same time, the oral administration of BMSCT has attracted much attention, because of its convenience, relative safety, and its ability to avoid systemic inflammatory reactions. It was found that the gut microbiota affects the development and treatment of cancer, and oral probiotics can regulate intestinal flora to achieve the purpose of treating the tumor. Shi et al. made an important discovery: specific members of the intestinal flora such as *bifidobacteria* could preferentially colonize at the tumor site and improve local anti-CD47 immunotherapy through stimulating interferon genes signaling at tumor sites. Furthermore, local delivery of *bifidobacterium* potently improves the cross-presentation in dendritic cells and induces the antitumor immunological effect of the host, thereby connecting adaptive and innate immunity in anti-CD47 immunotherapy [82]. However, due to the acidic and enzyme-rich digestive tract environment, oral probiotic delivery has encountered great challenges. It has been found that probiotics such as *Bacillus coagulans* are encapsulated in the biocompatible materials cationic polysaccharide chitosan and anionic polysaccharide alginate, which could enhance transportation, attachment and growth on the surface of the gastrointestinal tract [83]. Another biological interface supramolecular self-assembly method also described that probiotics were vortexed in calcium phosphate buffer with oleoylphosphatidic acid and cholesterol to coat the lipid membrane on the surface of probiotics. After oral administration, EcN with the lipid membrane could also effectively reach the gastrointestinal tract and maintain good activity [84]. By applying special biological materials to the surface of probiotics, probiotics can fight diseases more effectively. For example, the surface of the antitumor bacterium EcN was wrapped with erythrocyte membrane, which has good tumor-homing ability, low side effects, and good activity, so it has broad prospects in the tumor diagnosis and treatment [85].

Oral bacteria, as one type of antitumor drugs delivery carriers, have become a hot area of research because they can enhance the antitumor effects and reduce side effects. Mostaghaci et al. designed an oral bacterial robotic delivery approach, that is, *E. coli* connected with drug-containing nanoparticles (NPs) through a biotin–streptavidin bond. In the bacterial robot, the type I fimbriae of *E. coli* carrying lectin molecules have a great affinity to highly expressed mannose molecules on the surface of epithelial cells in the urethra and intestine. With this bioadhesive characteristic of the bacterial robot, the local concentration of the drug released from NPs at the desired site could be increased [86]. Oral bacteria could also be used to deliver biomacromolecules such as DNA vaccines or proteins. For instance, DNA-encapsulated cationic polymer-based NPs were modified on the surface of attenuated bacteria to form hybrid bacteria. After oral administration, hybrid bacteria could be absorbed into the blood and driven to the tumor site to activate the immune system [28]. In addition, Zhang et al. reported a light heat-controlled drug delivery system in which gold NPs were modified with heat-sensitive TNF-α-expressing engineered bacteria. The engineered bacteria could reach the tumor site through the gastrointestinal tract. After irradiating the tumor site with near-infrared (NIR) light, gold NPs could induce the expression of TNF-α and effectively kill the tumor cell [87].

**Fig. 10:**
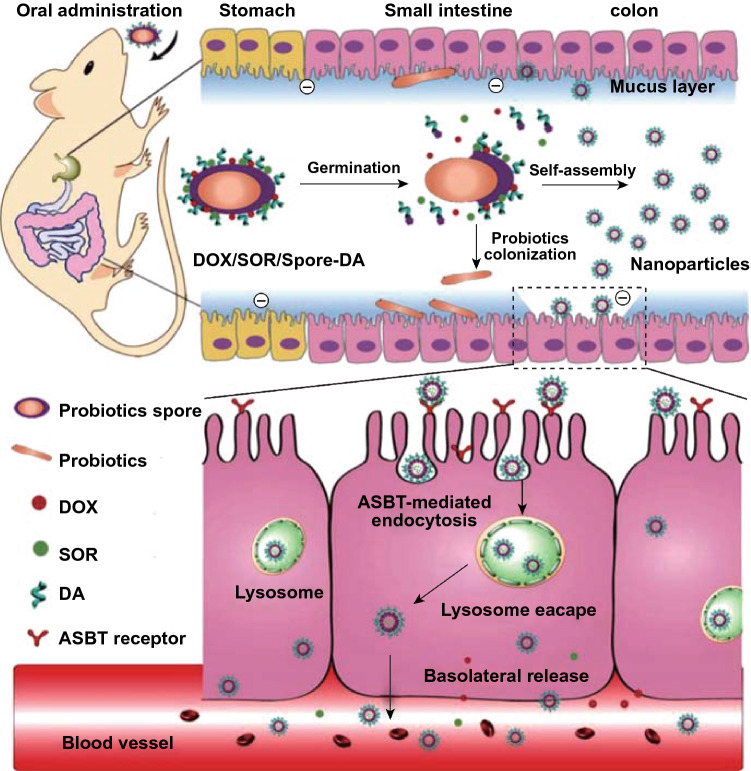
Schematic illustration of the Spore-Based Oral Autonomous Nanoparticles and its transepithelial transport mechanism. The chemotherapeutic drugs DOX and sorafenib (SOR), which are used as a synergistic treatment of cancer, are co-loaded in spores to construct an autonomous NPs generator (DOX/SOR/Spore-DA). The generator can continuously produce large amounts of DOX/SOR/Spore-DA NP in the GIT microenvironment. Subsequently, NP is effectively taken up by epithelial cells through the apical sodium-dependent bile acid transporter-mediated endocytic pathway, which can overcome various biological obstacles of the intestinal epithelium and increase the release of basolateral drugs, thereby improving the bioavailability of drugs. Reprinted with permission from Ref. [88]

Enclosing drugs in spores is also an effective strategy for improving drug bioavailability. A spore is a dormant bacterial body and can tolerate the acidic enzyme-rich digestive tract environment and germinate in the digestive tract. Song et al. modified deoxycholic acid (DA) on the surface of spores loaded with a chemotherapy drug, DOX, to form antitumor NPs. The NPs could improve the stability of DOX and overcome multiple biological barriers, thereby increasing the concentration of DOX in the tumor site (Fig. 10) [88]. Except for modification on the bacterial surface, chemotherapeutic drugs could be also encapsulated within the bacteria to prevent them from being destroyed by digestive juices and to improve drug absorption. In one study, cabazitaxel-containing lipid hybrid NPs were encapsulated into hollow yeast cells [89]. Similarly, Zhou et al. encapsulated several charged drugs in hollow yeast, and the bioavailability of drugs was significantly improved after oral administration. Hollow yeast filled with drugs could be internalized by M cells in the intestinal tract and engulfed by macrophages into the lymphatic system to further accumulate at the site of inflammation or tumors [90].

### Other Administration Routes of BMSCT

**Fig. 11:**
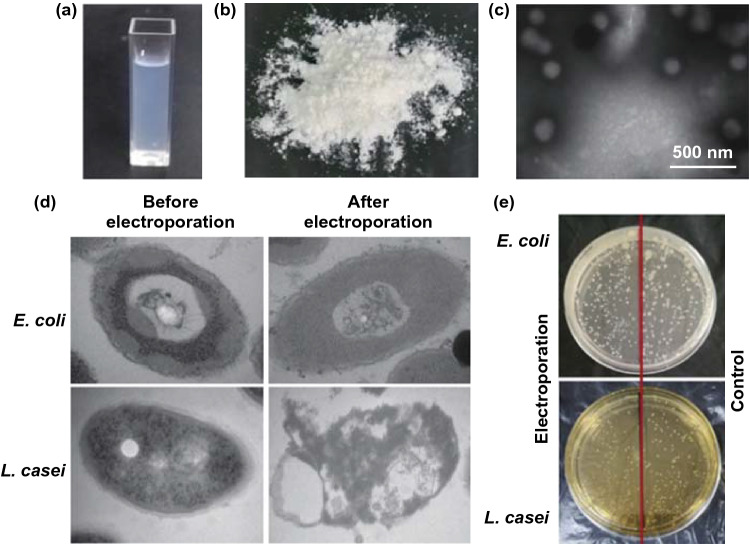
Characterization of LP-in-*E. coli* and LP-in-*L. casei*. **a** Appearance of liposomal paclitaxel (LP). **b** Appearance of LP powders. **c** TEM image of the rehydrated of LP powders. **d** TEM images of *E. coli* and *L. casei* before and after electroporation. **e** Comparison *of E. coli* and *L. casei* colonies with and without electroporation on the petri dishes. Reprinted with permission from Ref. [92]

Lung cancer is now responsible for 18.4% of deaths in all patients with malignancies worldwide [91]. The chemotherapeutic drugs administered via oral/intravenous routes induce severe toxic reactions because of non-targeting distribution in vivo. Inhalation is an important route of administration, which delivers the drugs directly to pathological sites in the lung tissue. Inhalation of bacterial formulations shows a significant advantage and would improve the effect of treating lung cancer. Zhang et al. reported that paclitaxel liposomes (LP) were contained in electroporated bacteria (*E. coli* or *L. casei*) to form LP-in-*E. coli* (LPE) or LP-in-*L. casei* (LPL). After inhalation administration, the drug accumulated in the lungs and effectively exerted anticancer effects with fewer side effects (Fig. 11) [92].

Transdermal administration, as an attractive topical approach, could achieve a desired treatment effect against melanoma, which is an aggressive cancer and located at the interface of the epidermis and dermis. It has been reported that bacteria-derived OMVs have a great potential to penetrate the stratum corneum (SC) and accumulate in the dermis, which could serve as a transdermal nanoplatform against melanoma [93]. Recently, Peng et al. proposed an OMV-based cocktail therapy for the treatment of melanoma. In this therapy, the α_ν_β_3_ integrin targeting ligand and ICG were modified on the surface of *E. coli* OMVs containing the tumor necrosis factor related apoptosis inducing ligand (TRAIL) gene. The modified OMVs could go through the stratum corneum and target the melanoma sites. After infrared light exposure, the OMVs would induce the production of reactive oxygen, and more important, they could activate TRAIL-induced apoptosis and trigger the release of TRAIL to enhance the anticancer effect. In addition, this nanoplatform could treat some other skin diseases with the aid of bacteria-derived OMVs [93].

There are various challenges in the bacteria-mediated transdermal therapy, including: (1) it is difficult for biological macromolecules to pass through the stratum corneum, (2) a sufficiently high drug concentration is required at the tumor site to ensure an excellent antitumor effect, and (3) the safety of the transdermal drug delivery system must be ensured. As we know, the stratum corneum has formed a great barrier, preventing the penetration of external biological macromolecules. The complicated use of physical devices such as electroporation makes its clinical application challenging [94]. Bacteria cannot penetrate the stratum corneum and enter the body under normal circumstances. However, bacteria-derived OMVs with nanometer sizes have great potential to penetrate the stratum corneum. Studies have reported that OMV with a diameter of 361.28 nm could successfully penetrate the stratum corneum and accumulate in the dermis [95]. Meanwhile, transdermal drug delivery systems need to have good tumor targeting to ensure that the drug is well-distributed in the body, that is, the concentration of drug should be high in the tumor site but low in normal tissues and organs. To achieve this, some tumor-targeting ligands, such as α_ν_β_3_ integrin, could be modified on the surface of OMVs so that drug-loaded OMVs can accumulate at the tumor site. The important thing is that OMVs must be non-pathogenic, good biocompatibility and low immunogenicity [93].

In the transdermal therapy, intact bacteria are not suitable as the carrier, because it is difficult to penetrate the stratum corneum. Nano-scale OMVs with the ability of penetrating the stratum corneum could be used as a delivery vehicle for transdermal delivery. When choosing OMVs, the safety should be the first consideration. Pathogenic OMVs entering the body might cause acute inflammation or even sepsis. To ensure safety of transdermal therapy, we can choose OMVs from attenuated bacteria or probiotics such as *E. coli*. Moreover, we should also consider the size of OMVs, and OMVs with less than 360 nm might be suitable for application in transdermal therapy. In addition, the drug loading capacity of OMVs and tumor targeting are also important [94] (Table 2).

**Table 2:** Summary of bacteria-mediated synergistic cancer therapy

Synergistic bacteria-mediated therapy	Administration route	Strain (s)	Reagent (s)	Type of tumor	References
Chemotherapy	Peritumorally injected	*Magnetococcus marinus* strain MC-1	SN-38	HCT116	[38]
Chemotherapy	Intravenous injection	*E. coli* Nissle 1917	DOX	4T1	[39]
Chemotherapy	Intravenous injection	*E. coli* Nissle 1917	DOX, α-tocopheryl succinate	4T1	[41]
Chemotherapy	Intravenous injection	*S. typhimurium* minCDE–	SiRNA, irinotecan	colon cancer	[45]
Photothermal therapy	Intravenous injection	*S. typhimurium* strain YB1(YB1)	ICG	MB49	[48]
Photothermal therapy	Intravenous injection	*Salmonella* strain VNP20009(VNP20009)	Polydopamine	B16-F10	[49]
Photothermal therapy	Intravenous injection	*S. oneidensis* MR-1	Palladium nanoparticles, methylene blue	B16-F10	[51]
Photothermal therapy	Intravenous injection	*C. difficile* CCUG 37780	Upconversion nanorods, Au nanorods	A549	[52]
Photothermal therapy	Intratumoral injection	*E. coli* K12	Melanin	4T1	[53]
Photothermal therapy	Footpad injection	*Salmonella*	Poly(lactic-co-glycolicacid)–indocyanine green	B16-F10	[55]
Photothermal therapy	Intravenous injection	*E. coli* Trans T1	Polymer PBIBDF-BT (with alternating isoindigo derivative and bithiophene)	EMT6, CT26	[58]
RNS therapy	Intravenous injection	*E. coli* MG1655	Carbon nitride	4T1, CT26	[62]
ROS therapy	Intravenous injection	*E. coli* MG1655	Fe_3_O_4_	CT26	[63]
ROS therapy	Intravenous injection	*Synechococcus* 7942	HAS, ICG	4T1	[65]
ROS therapy		*E. coli*	TDNPP	HeLa	[66]
Immune therapy	Intravenous injection	*E. coli* TOP10		CT26	[67]
Immune therapy	Intratumoral injection	*E. coli* Pir1^+^		4T1, B16-F10, A20	[31]
Immune therapy	Intravenous injection	ΔppGpp.*S. typhimuriumstrain* SHJ2037		MC38, B16-F10	[68]
Immune therapy	Oral administration	*S. typhimurium*		CMS5a	[9]
Immune therapy	Intravenous injection	*E. coli* msbB^−/−^		CT26	[70]
Immune therapy	Intratumoral injection	*Mycobacterium smegmatis*	PC7A/CpG	B78 melanoma, NXS2 neuroblastoma	[71]
Immune therapy	Intravenous injection	*S.typhimurium*	Tegafur	B16-F10	[72]
Prodrug-activating therapy		*E. coli* BL21	P-hydroxy aniline mustard β-D-glucuronide	HCT116	[77]
Prodrug-activating therapy	Intravenous injection	*E. coli* DH5α-lux/βG	Glycyrrhizic acid	CT26	[80]
Immune therapy	Oral administration	*Bifidobacterium*		MC38	[82]
Immune therapy	Oral administration	*Bacillus coagulans*			[83]
	Oral administration	*E. coli* Nissle 1917			[84]
Immune therapy	Oral administration	*E. coli* Nissle 1917		4T1	[85]
	Oral administration	*E. coli* MG1655			[86]
Immune therapy	Oral administration	*E. coli* MG1655	TNF-α, Gold nanoparticles	4T1	[87]
Chemotherapy	Oral administration	*Bacillus cagulans*	DOX, SOR	SW620	[88]
Chemotherapy	Oral administration	Yeast Cell	Cabazitaxel	Raw 264.7	[89]
Chemotherapy	Oral administration	Yeast Cell	Indomethacin, Paclitaxel	MCF7	[90]
Chemotherapy	Pulmonary inhalation	*E. coli, L. casei*	Paclitaxel	A549	[92]
Photothermal therapy	Transdermal delivery	*E. coli*	ICG	B16-F10	[93]

## Challenges and Future Prospective

Although many published studies on bacteria-based bio-therapy have shown promising prospects in the experimental models, substantial obstacles remain for using tumor-targeting bacteria in clinical practice as therapeutic agents. Firstly, safety is the major concern because of the infectious nature of the bacteria. BMSCT is a deliberate attempt to convert the tumor tissue into a localized tumor-destructing infection. This strategy would have serious consequences once the infection spread over the whole body. Effective management of therapeutic infection, timely antibiotic intervention and choice of safe bacterial strains, could improve the control of bacterial toxicity. In addition, live bacteria carrying antibiotic resistance genes that can cause serious infections are generally not suitable for clinical applications. Chromosomal integration of the heterologous DNA without antibiotic selection plasmids provides a practical way to ensure the bio-therapy safety [96]. Secondly, limited drug loading efficiency is another challenge dampening the anticancer effect of bacteria. Except for further optimization of preparation methods, the genetic engineering of bacteria to produce anticancer agents might enhance the resulting therapeutic outcomes. Thirdly, the manufacturing process of live bacteria is more complex than that of the small molecule anticancer drugs. Unlike small molecules or other non-viable clinical agents, live therapeutic bacteria cannot be sterilized by filtering or heating, which would be the main challenge for producing Good Manufacturing Practices grade test products. The conventional standard for sterile test would not be applicable. Hence, producing, purifying, and harvesting live bacteria following strict aseptic protocols with real-time supervision are a practical way to ensure the quality of the final products. The bacterial seed stock and banking system should also be further optimized. Last but not least, when live bacteria could be used in a clinical setting, the potential impact on the environment would be also a concern that should be properly addressed.

Bacteria-mediated combination therapy must have broad applications in the future. At present, due to the heterogeneity of tumor at the molecular and histological level, cancers are difficult to cure with monotherapy. Bacteria can deliver a variety of drugs, amino acids, and proteins. The excellent tumor-targeting properties allow bacteria to play an important role in the treatment of cancer. However, there are also some problems in bacteria-mediated synergistic cancer therapy that need to be resolved in the future work. In order to promote bacterial synergy therapy into the clinic, researchers also need to invest more energy in researching large-scale production, sterilization technology, management plans, production equipment, storage and transportation methods.

## Conclusions

In summary, recent studies have shown that bacteria can anchor and inhibit the growth of tumors and even eradicate the tumors in some cases. However, the heterogeneity of tumors makes it difficult achieve a cure with single bacterium therapy. Thus, researchers have shifted to either using bacteria as effective vectors that shuttle chemotherapy agents, such as paclitaxel, 5-fluorouracil, small interfering RNA and doxorubicin hybrid micelles, as well as PSs like ICG and palladium nanoparticles into tumors or serve as excellent adjuvants for immunotherapy due to their capacity to sensitize the host immune system. To avoid safety concerns, another approach is to replace the bacterial vector by bacterial OMVs that can shuttle a range of chemotherapeutic drugs into tumors. This possibility has been rarely explored until now, but its great potential is notable. Furthermore, oral administration of bacterial preparations also has been employed for cancer treatment recently. However, the therapeutic efficacy is strongly hampered by the acidic gastric environment and abundant metabolic enzymes in the gastrointestinal tract, and further investigation is needed to modify bacterial preparations.

Taken together, the unique features of bacteria—that they specifically colonize tumor regions and induce tumor inhibitory responses—in combination with their potential as ideal drug delivery vehicles provide a solid platform for cancer treatment. From the first attempt to trace Coley’s strategy until today, great progresses has been achieved, and this progress will continue. Thus, with more rational design, bacterial therapy will grow into one of the most powerful weapons in the battle against cancers in the near future.
